# Study on the Importance of Hygienic Hand Disinfection of Dental Practitioners and Students as an Infection Control Measure in Dental Practice

**DOI:** 10.3390/antibiotics15020205

**Published:** 2026-02-13

**Authors:** Veselina Kondeva, Velina Stoeva, Yordan Kalchev, Rumyana Stoyanova

**Affiliations:** 1 Department of Pediatric Dentistry, Faculty of Dental Medicine, Medical University of Plovdiv, 4002 Plovdiv, Bulgaria; veselina.kondeva@mu-plovdiv.bg; 2 Department of Epidemiology and Disaster Medicine, Faculty of Public Health, Medical University of Plovdiv, 4002 Plovdiv, Bulgaria; 3 Department of Medical Microbiology and Immunology, Faculty of Medicine, Medical University of Plovdiv, 4002 Plovdiv, Bulgaria; yordan.kalchev@mu-plovdiv.bg; 4 Division of Innovative Diagnostic Methods, Research Institute at Medical University of Plovdiv, Medical University of Plovdiv, 4002 Plovdiv, Bulgaria; 5 Department of Health Management and Health Economics, Medical University of Plovdiv, 4002 Plovdiv, Bulgaria; rumyana.stoyanova@mu-plovdiv.bg

**Keywords:** hand antisepsis, healthcare associated infections, prevention of infections, dentistry, dental practice

## Abstract

The hands of dental students and practitioners are a key epidemiological factor in the transmission of infections associated with dental care. Strict adherence to the established hand hygiene protocols, combined with regular training and monitoring the quality of the performed hygienic hand disinfection, is crucial for ensuring safe dental practice. **Objectives**: The aim of the study is to assess the quality of hand antisepsis performed with alcohol-containing preparation among dental students and practicing dentists. **Methods**: A prospective epidemiological and microbiological study was conducted on 225 people—149 students from the 4th, 5th and 6th year of training at the Faculty of Dental Medicine, Plovdiv, and 76 dentists. The skin antiseptic was applied according to the “six steps” method with alcohol-based antiseptics. The samples were taken with a dry sterile swab. **Results**: The comparison between students and practicing dental medical doctors shows that the latter have a higher relative share of samples with microbiological growth 12 (15.8%), including coagulase-negative staphylococci (CoNS) above 10^5^ CoNS, compared to students 6 (4.0%), (*p* = 0.004). Gram-negative microbiological isolates indicate a statistically significant gender dependence (*p* = 0.016)—15 in men (15.8%), compared to 7 in women (5.4%). Growth of fungi (yeasts and mols) is statistically significant depending on gender (*p* = 0.015) and is observed only in men. **Conclusions**: The presence of significant microbial counts of CoNS is an indicator of insufficiently effective hygienic hand disinfection. The recovery of Gram-negative enteric bacteria is unacceptable and suggests serious shortcomings in the hygienic disinfection of some of the samples studied. Students demonstrated superior hand antisepsis performance compared to practicing dentists.

## 1. Introduction

The hands of dental personnel represent a major factor in the transmission of microorganisms in dental practice, and their hygienic disinfection is a fundamental element in controlling healthcare-associated infections. It is widely recognized as the most effective measure for interrupting the transmission of microorganisms between patients and dental personnel, and vice versa. This is because the hands of dental team members are in constant and direct contact with blood, saliva, oral fluids, as well as instruments, equipment, and surfaces contaminated with them, which determines their role as a key vector in cross-infections [1].

The use of alcohol-based disinfectants combined with correct performance of hygienic hand disinfection has been shown to significantly reduce transient microflora and limit the spread of pathogenic microorganisms in the dental clinical environment [2,3]. International recommendations for the prevention of healthcare-associated infections are clear and unambiguous and should be consistently applied in clinical practice [4]. However, several studies have reported insufficient knowledge and, consequently, suboptimal compliance with hand hygiene measures among dentists and dental students [5,6,7].

Some studies indicate that many trainees and students have an inadequate understanding of the level of protection provided by gloves, which may result in improper glove use [8]. Other surveys have shown that a proportion of dental students and dentists incorrectly believe that gloves provide complete protection and therefore do not always change them during prolonged procedures [9]. These findings suggest that inadequate glove use and infrequent glove replacement may contribute to compromised infection control practices.

More recent studies have examined similar shortcomings in the implementation of hygienic hand disinfection among dental students and indicate that this problem remains relevant. Despite awareness of the importance of hand hygiene, students demonstrate limited knowledge of other infection control measures. Commonly reported reasons for insufficient hand hygiene include lack of time, high workload, and limited access to appropriate handwashing facilities [10].

Similarly, suboptimal compliance has been reported in a study conducted in a teaching dental hospital. The study documented low baseline hand hygiene compliance (~15.6%) among healthcare workers, including trainees, which increased only modestly after targeted training interventions (to approximately 36%). Notably, the lowest compliance rates and the smallest improvements were observed among practicing dentists, highlighting persistent gaps in routine clinical practice [11].

A team of authors investigated the implementation of handwashing among dental students after the pandemic period to determine the extent to which students apply what they have learned about hand hygiene in their clinical practice and whether trends have changed after COVID-19. The authors concluded that, despite the impact of the COVID-19 pandemic, handwashing is not a standard practice among dental students and that it is imperative to implement educational measures encouraging true adherence to hand hygiene practices [12].

In addition, the use of high-speed dental equipment during most dental procedures increases the risk of aerosol generation, facilitating long-distance dispersion and contamination of the dental environment with bacteria and viruses, including SARS-CoV-2. This further underscores the importance of effective hygienic hand disinfection as a preventive measure against both direct and indirect transmission of healthcare-associated infections. In this context, strict adherence to established hand hygiene protocols, combined with regular training and monitoring of hand disinfection quality among dental staff and students, is essential for ensuring safe dental practice and protecting both patients and dental professionals [13,14,15,16,17].

The study aimed to assess the quality of hand antisepsis performed with an alcohol-containing preparation before performing a prophylactic or therapeutic procedure on a patient, among dental students and practitioners.

The study was conducted under the following null hypotheses (H_0_):

**H_01_**: *There is no statistically significant difference in microbiological growth after hygienic hand disinfection between dental students and practicing dentists.*

**H_02_**: *There is no statistically significant association between gender and microbiological growth after hygienic hand disinfection.*

**H_03_**: *There is no statistically significant association between year of study or professional experience and microbiological growth after hygienic hand disinfection.*

A *p*-value < 0.05 was considered statistically significant for rejection of the null hypotheses.

## 2. Materials and Methods

### 2.1. Procedure

A prospective epidemiological and microbiological study was conducted on 225 people—149 students and 76 dental practitioners. Dental students from the Faculty of Dental Medicine, Plovdiv, from the 4th, 5th and 6th years, were studied in the period September–November 2025. The participating dentists from the city of Plovdiv were studied in the period October–November 2025.

The skin antiseptic was applied according to the established standards for quantity and exposure using the “six steps” method. The samples were taken with a dry sterile swab through hand washing in order to reach the areas often missed during hygienic disinfection (between the fingers, fingertips, back of the thumb, etc.).

**Criteria for inclusion in the study:** Fourth, 5th and 6th year dental students studying at the Faculty of Dental Medicine–Plovdiv and respectively having clinical work with patients (clinical practice with patients starts from the 4th year and this determines the selection of these groups of students) and practicing dentists from the city of Plovdiv.

**Exclusion criteria:** all those who do not belong to the groups mentioned above.

Participants fill out an informed consent form in advance, which explains all aspects of the study, its objectives and scientific benefits, and are assured that the results will be used only for scientific purposes and only in a summarized form. Those who participate in the study have the opportunity to ask questions orally to the person conducting the sampling. By filling out the informed consent form provided to them, they declare their voluntary consent to participate in the study.

Sampling of students was carried out in the clinical rooms in the presence of their teachers and the dental nurse of the respective clinical room during their practical exercises with patients, before performing a prophylactic or therapeutic manipulation.

Sampling of dentists was carried out in their dental offices before performing a prophylactic or therapeutic manipulation, at a time convenient for them within their working day.

### 2.2. Microbiological Analysis

Microbiological evaluation included screening for facultative anaerobic and aerobic microorganisms, serving as an indicator to assess the performed decontamination practices. Samples for microbiological analysis were collected with dry sterile cotton swabs, with transport medium (Amies, Biolife Italiana S.r.l., Milan, Italy). The participants‘ hands were swabbed using firm and consistent pressure with the swab being rotated continuously while sampling and moved in a zig-zag or circular motion to maximize bacterial recovery. Swabbing was focused on critical areas prone to suboptimal disinfection including the palm, interdigital space, thumb, and beneath the fingernails. Immediately after collection the swabs were placed in the corresponding transport medium.

Collected samples were inoculated no later than 24 h after collection. Inoculation was performed on 5% sheep blood agar (Diachim, Sofia, Bulgaria), eosin-methylene blue agar (Diachim, Sofia, Bulgaria) and Candida chromogenic agar (Diachim, Sofia, Bulgaria). The blood agar and eosin-methylene blue plates were incubated at 37 °C for a maximum period of 48 h. The blood agar was used to screen for Gram positive bacteria, whereas the eosin-methylene blue agar was used to screen for Gram-negative bacteria. The Candida chromogenic media used for screening for fungi were incubated for a maximum of 5 days. For broad bacterial identification, we used routine manual biochemical tests (catalase, coagulase, oxidase, esculin hydrolysis test, and arabinose fermentation).

### 2.3. Data Analysis

Descriptive statistical methods and non-parametric statistical tests were applied for the processing and analysis of the empirical data. Qualitative variables are presented as absolute frequencies and relative proportions (percentages), allowing for a clearer visualization of the structure of the studied sample and facilitating comparative analysis.

To assess associations between categorical variables, the Pearson chi-square test was used for multivariate contingency tables, while Fisher’s exact test was applied for two-dimensional 2 × 2 tables.

The level of statistical significance was set at *p* < 0.05, indicating that the probability of the observed results occurring by chance is less than 5%, thereby allowing rejection of the null hypothesis with an adequate level of confidence.

The data were statistically processed using the software products SPSS 23 and MS Excel 2016.

### 2.4. Ethical Considerations

The study was approved by the Ethics Committee of the Medical University of Plovdiv with a protocol dated 24 September 2025.

## 3. Results

### 3.1. Students

The gender distribution of the surveyed students was as follows: 41.6% (n = 62) were men and 58.4% (n = 87) were women. The distribution by year of study was relatively even: 32.9% (n = 49) were in their fourth year, 32.9% (n = 49) in their fifth year, and 34.2% (n = 51) in their sixth year.

The most significant results regarding the microbiological isolates are presented below. On blood agar, no microbial growth was observed in 75.2% (n = 112) of the samples; 20.8% (n = 31) showed growth below 10^5^ (colony-forming unit) CFU of coagulase-negative staphylococci (CoNS), while 4.0% (n = 6) demonstrated growth at least 10^5^ CFU of CoNS (Table 1).

On eosine-methylen blue agar, in 91.3% (n = 136) no growth of microorganisms was recorded, but in 8.7% (n = 13) there was growth below 10^5^ Gram-negative enteric bacteria, and in none was growth above 10/5CoNS recorded (Table 2).

On Candida chromogenic agar, 98.7% (n = 147) did not show growth of fungi, but 1.3% (n = 2) showed growth (Table 3). All fungi were detected in less than 10^5^ CFU.

Statistical associations were examined between gender, year of study, and the presence and intensity of microbiological growth.

#### 3.1.1. Relationship Between Gender and Microbiological Growth

Analysis of the association between gender and microbiological growth on blood agar (Table 1) revealed no statistically significant differences. Among men, no growth was recorded in 67.7% (n = 42), growth below 10^5^ CoNS was observed in 29.0% (n = 18), and growth above 10^5^ CoNS in 3.2% (n = 2). Among women, absence of growth was found in 80.5% (n = 70), growth below 10^5^ CoNS in 14.9% (n = 13), and growth above 10^5^ CoNS in 4.6% (n = 4). These differences did not reach statistical significance (χ^2^ test, *p* = 0.111), indicating that gender did not significantly influence the presence or intensity of microbiological growth on blood agar.

In contrast, analysis of the relationship between gender and microbial growth on eosin-methylen blue agar (Table 2) demonstrated a statistically significant difference. Among men, no growth was recorded in 85.5% (n = 53) of the samples, while in 14.5% (n = 9) growth below 10^5^ Gram-negative enteric bacteria was observed. Among women, absence of growth was significantly more frequent, occurring in 95.4% (n = 83) of the samples, whereas growth below 10^5^ Gram-negative enteric bacteria was observed in only 4.6% (n = 4). The difference was statistically significant (Fisher’s exact test, *p* = 0.042), indicating a higher frequency of contamination with microorganisms growing on eosine-methylen blue agar among men.

The analysis of Candida chromogenic agar (Table 3) did not show a statistically significant dependence on gender. In men, no microbial growth was detected in 96.8% (n = 60) of the samples, while fungal growth below 10^5^ was observed in 3.2% (n = 2). In women, no microbiological growth was detected in any of the samples (100%, n = 87). The observed differences were not statistically significant (Fisher’s exact test, *p* = 0.172).

#### 3.1.2. Relation Between Training Courses and Growth of Microorganisms

Analysis of microbiological growth on blood agar according to the year of study (Table 1) revealed a statistically significant difference (*p* = 0.021). Among fourth-year students, 81.6% (n = 40) did not show growth, 14.3% (n = 7) reported growth below 10^5^ CoNS, and 4.1% (n = 2) reported growth above 10^5^ CoNS. In fifth-year students, the lack of growth was most common—85.7% (n = 42), followed by growth below 10^5^ CoNS in 12.2% (n = 6) and above 10^5^ CoNS in 2.0% (n = 1). The highest proportion of samples with microbiological growth was observed among sixth-year students, in whom 58.8% (n = 30) had no growth, 35.3% (n = 18) had growth below 10^5^ CoNS, and 5.9% (n = 3) had growth above 10^5^ CoNS.

Regarding eosine-methylen blue agar (Table 2), a statistically significant dependence on the course of study was also found (*p* = 0.001). In the fourth-year students, no growth was detected in 79.6% (n = 39), while in 20.4% (n = 10), growth below 10^5^ Gram-negative enteric bacteria was detected, which represents the highest relative proportion of contamination. In the fifth-year students, no microbiological growth was detected in all samples tested (100%, n = 49). Among the sixth-year students, the lack of growth was reported in 94.1% (n = 48), and growth below 10^5^ Gram-negative enteric bacteria—in 5.9% (n = 3).

Analysis of microbiological growth on Candida chromogenic agar depending on the course of study (Table 3) did not show a statistically significant difference (*p* = 0.126). In the fourth- and sixth-year students, no growth was detected in any of the samples, while in the fifth-year students, 95.9% (n = 47) lacked growth, and in 4.1% (n = 2) the presence of microorganisms was observed.

### 3.2. Dentists

The gender distribution of the studied dentists was as follows: 43.4% (n = 33) were men and 56.6% (n = 43) were women. Regarding professional experience, 28.9% (n = 22) had up to 5 years of experience, 38.2% (n = 29) had between 5 and 15 years of experience, and 32.9% (n = 25) had more than 15 years of experience.

The analyses of the microbiological isolates in the studied group of dentists are presented in Table 4 (blood agar), Table 5 (eosin-methylene blue agar) and Table 6 (chrome agar).

As shown in Table 4, Table 5 and Table 6, the majority of dentists did not exhibit microbiological growth on the culture media used. When growth was detected, it was predominantly of low intensity, with only isolated cases demonstrating a higher microbiological load.

Statistical analysis using the chi-square test did not reveal statistically significant associations between gender and microbiological growth, nor between professional experience and microbiological growth on blood agar, eosin-methylene blue agar, and Candida chromogenic agar in the studied group (*p* > 0.05).

### 3.3. Comparative Results for Students and Working Dentists

The overall gender distribution of the combined sample was 42.2% (n = 95) men and 57.8% (n = 130) women. With regard to group allocation, 66.2% (n = 149) of the participants were students, while 33.8% (n = 76) were practicing dentists.

The analyses of the microbiological isolates in the two studied groups are as follows: students and dentists are presented in Table 7 (blood agar), Table 8 (Levin) and Table 9 (chrome agar).

The data presented in Table 7 illustrate the distribution of microbiological growth on blood agar according to gender and group affiliation. With respect to gender, no statistically significant difference was observed in either the frequency or intensity of microbiological growth (*p* = 0.096). In contrast, comparison between students and practicing dentists revealed a statistically significant difference (*p* = 0.004), with the group of dentists demonstrating a higher relative proportion of samples with microbiological growth, including growth above 10^5^ CoNS, compared to students.

Analysis of the microbiological isolates on eosin-methylene blue agar (Table 8) demonstrated a statistically significant association with gender (*p* = 0.016). Microbiological growth was more frequently observed in men, whereas samples without growth predominated among women. Conversely, no statistically significant difference was identified between students and practicing dentists in terms of the growth of eosin-methylene blue agar microorganisms (*p* = 0.277).

The results presented in Table 9, which describe microbial growth on Candida chromogenic agar, also revealed a statistically significant difference according to gender (*p* = 0.015). Growth of yeast and mold was detected exclusively in men, while no microbiological growth was observed in women. Comparison between students and practicing dentists did not demonstrate a statistically significant difference (*p* = 0.107), although isolated cases of yeast and mold growth were recorded among dentists.

## 4. Discussion

The hands of dental staff and students represent a major vector for the transmission of microorganisms between patients, dental personnel, clinical surfaces, equipment, and instruments. Inadequately performed hygienic hand disinfection increases the risk of persistence of both resident and transient microbiota, and their effective removal is essential for the prevention of healthcare-associated infections [18].

Numerous microbiological studies investigating hand contamination among dental professionals and students report findings comparable to those observed in the present study. Coagulase-negative staphylococci (CoNS) are consistently among the most frequently isolated microorganisms, even after hygienic hand disinfection. According to the literature, the most commonly detected species include *Staphylococcus epidermidis* and *Staphylococcus hominis*, while *Micrococcus luteus* is also frequently identified. Although these microorganisms are typical components of the normal skin microbiota, they may act as opportunistic pathogens, particularly in immunocompromised patients [19].

Importantly, CoNS may serve as an indirect indicator of the quality of hand antisepsis. In the present study, CoNS predominated among the isolated microorganisms. While their presence alone does not necessarily indicate a pathogenic risk, their detection in high microbial loads following hygienic hand disinfection suggests significant shortcomings in the execution of antiseptic procedures. Similar observations have been reported in the literature, where the persistence of CoNS after disinfection has been attributed to inadequate technique and/or improper application of alcohol-based antiseptics. Pittet et al. emphasize that insufficient contact time, particularly when using alcohol-based formulations, may prevent the achievement of the logarithmic reduction required for effective microbial control [20].

Another important factor contributing to ineffective hand antisepsis is the insufficient volume of disinfectant applied. For optimal antimicrobial efficacy, all skin surfaces must be evenly and thoroughly wetted [21]. Residual microbial contamination is most frequently detected in areas that are often neglected during hand disinfection, including the subungual spaces, interdigital areas, fingertips, and the dorsal surface of the thumb [18,22]. These considerations informed our choice of sampling method using dry sterile cotton swabs, which allowed effective sampling of these high-risk areas.

Current guidelines issued by the Centers for Disease Control and Prevention (CDCs) and the World Health Organization (WHO) consistently emphasize the importance of correct scrubbing technique, adequate duration of hand antisepsis, appropriate selection of antiseptic agents, and proper skin care. The persistence of CoNS following hand disinfection should therefore not be underestimated but rather interpreted as a clinically relevant marker of the effectiveness of infection control practices in dental settings [4,18].

*Staphylococcus aureus* is another microorganism frequently isolated from the hands, oral cavity, and nasal mucosa of both dental students and patients, and its presence is associated with an increased risk of cross-infection during clinical training. Previous studies have linked *S. aureus* colonization to improper glove use and contamination of the dental clinical environment [23,24]. In comparative investigations evaluating hand contamination among dentists with and without rings, *S. aureus*, *Escherichia coli*, and *Candida albicans* were isolated more frequently in individuals wearing rings under their gloves. These findings highlight that even when routine cleaning is performed, hands may remain contaminated with potentially pathogenic microorganisms if antiseptic procedures are inadequately executed [25].

In the present study, *S. aureus* was not detected in any of the examined samples, which may be considered an indicator of satisfactory hand hygiene, as its presence is commonly associated with insufficient disinfection.

Several microbiological investigations have reported the detection of enteric Gram-negative bacteria (*Enterobacterales*) and opportunistic fungi, such as *Candida* spp., on the hands, gloves, and work clothing of dental professionals and students [25,26]. Although detected in a relatively small proportion of samples, the presence of Gram-negative enteric bacteria in our study is unacceptable from an infection control perspective and suggests non-compliance or misunderstanding of standard precautions. Potential contributing factors include inadequate handwashing or disinfection techniques, wearing rings or other jewelry beneath gloves, and failure to adhere to appropriate glove-changing protocols between patients [4,18].

Behavioral studies further support these microbiological findings. A review of the literature reported that female healthcare workers generally demonstrate better hand hygiene practices than their male colleagues [27]. In line with these observations, our results revealed a statistically significant association between gender and microbiological growth on eosin-methylene blue agar, with contamination being more prevalent among male participants.

Recent studies have also highlighted persistent deficiencies in hygiene practices among healthcare students. AlNaser et al. examined hand and smartphone contamination among medical and dental students and found no significant differences in bacterial colony counts on the hands, despite lower contamination of smartphones among medical students. The authors emphasized the importance of comprehensive training in hand hygiene and routine disinfection of personal devices to minimize the risk of cross-contamination [28]. Similarly, a systematic review and meta-analysis by Resende et al. demonstrated that less than 50% of hand hygiene opportunities among dental students in academic institutions were adequately performed, underscoring the need for sustained educational interventions [29].

In addition to gender-related differences, our findings demonstrate that the year of study may be associated with variations in microbiological growth. Statistically significant associations were observed on blood agar and Levin’s medium, indicating that clinical exposure, intensity of practical training, and adherence to anti-epidemic measures may influence the effectiveness of hygienic hand disinfection. The higher proportion of microbiological growth observed among sixth-year students may be explained by their more frequent contact with patients and biological materials during advanced clinical training. In contrast, the most favorable results were recorded among fifth-year students, possibly reflecting an optimal balance between theoretical preparation and supervised clinical practice. The relatively higher contamination observed among fourth-year students may be attributed to limited clinical experience and less established infection control routines.

No statistically significant differences were observed on chromogenic agar, suggesting that fungal contamination was not specifically associated with the year of study and that effective control of these microorganisms was generally maintained across all student groups. Among practicing dentists, no significant association was found between years of professional experience and microbiological growth across the different culture media, indicating that prolonged professional experience alone does not necessarily translate into better hand hygiene performance.

The finding that practicing dentists demonstrated higher levels of hand contamination compared to students raises important concerns regarding the adequacy of postgraduate education and ongoing professional training in infection prevention. These results contrast with some reports indicating better compliance among practicing dentists than among students [29] and highlight the need for continuous monitoring and reinforcement of hand hygiene practices in daily clinical work.

In conclusion, systematic training, regular assessment of hand hygiene performance, and strict control of compliance with infection prevention protocols are essential components in the reliable prevention of dental healthcare-associated infections. These measures should be integral to undergraduate dental education, while continuing professional development should ensure that infection control knowledge is regularly updated and effectively translated into routine clinical practice, reflecting both individual and public health responsibility [30,31,32].

We should address some limitations of the presented study. Firstly, the participants performed their hand disinfection by alcohol-based hand-rubs, all of which approved for clinical use. However, it is important to note that different brands were used by the participants, which might have caused some variability in the hand disinfection and the subsequent bacterial recovery. Secondly, another limitating factor of this study is the lack of control over individual hand characteristics, such as skin dryness or oiliness. Variations in skin condition and environmental exposure may influence bacterial adherence and growth, potentially affecting the results. To minimize the impact of this limitation, bacterial counts equal to or exceeding 10^5^ CFU were considered indicative of a significant bacterial load.

## 5. Conclusions

Although coagulase-negative staphylococci, which belong to the resident skin microbiota, are the main isolates in most samples, their presence in high microbial numbers is undesirable. This could be significant for immunosuppressed patients and is also an indicator of insufficiently effective hygienic hand disinfection. Gram-negative intestinal bacteria were also isolated, which is unacceptable and indicates a serious misunderstanding or non-compliance with hand antisepsis protocols.

This study demonstrated that students exhibited better hand antisepsis performance than practicing dentists. While this likely reflects the impact of undergraduate infection control education, it also indicates that sustained compliance with hygiene protocols in clinical settings may diminish over time as a result of time pressures, habitual behaviors, and the absence of routine monitoring. Thus, the obtained suboptimal results point us to the need to increase the knowledge and commitment of dental personnel in the decontamination of hands before manipulations.

Training and control of knowledge on decontamination, disinfection, proper use of personal protective equipment, etc., are key elements in the prevention of dental care-associated infections.

## Figures and Tables

**Table 1:** Blood agar microbiological isolates in the student group.

Demographic Characteristics		Blood Agar			Total n (%)	*p*
		No Growth n (%)	Growth < 10^5^ CoNS n (%)	Growth ≥ 10^5^ CoNS n (%)		
Sex	Male	42 (67.7%)	18 (29.0%)	2 (3.2%)	62 (100.0%)	0.111
	Female	70 (80.5%)	13 (14.9%)	4 (4.6%)	87 (100.0%)	
Total		112 (75.2%)	31 (20.8%)	6 (4.0%)	149 (100.0%)	
Year of study	Fourth year	40 (81.6%)	7 (14.3%)	2 (1.3%)	49 (100.0%)	0.021
	Fifth year	42 (85.7%)	6 (12.2%)	1 (2.0%)	49 (100.0%)	
	Sixth year	30 (58.8%)	18 (35.3%)	3 (5.9%)	51 (100.0%)	
Total		112 (75.2%)	31 (20.8%)	6 (4.0%)	149 (100.0%)	

**Table 2:** Microbiological isolates on eosin-methylene blue agar in the student group.

Demographic Characteristics		Eosin-Methylene Blue Agar		Total n (%)	*p*
		No Growth n (%)	Growth < 10^5^ n (%)		
Sex	Male	53 (85.5%)	9 (14.5%)	62 (100.0%)	0.042
	Female	83 (95.4%)	4 (4.6%)	87 (100.0%)	
Total		136 (91.3%)	13 (8.7%)	149 (100.0%)	
Year of	Fourth year	39 (79.6%)	10 (20.4%)	49 (100.0%)	0.001
study	Fifth year	49 (100.0%)	0 (0.0%)	49 (100.0%)	
	Sixth year	48 (94.1%)	3 (5.9%)	51 (100.0%)	
Total		136 (91.3%)	13 (8.7%)	149 (100.0%)	

**Table 3:** Microbiological isolates on Candida chromogenic agar in the student group.

Demographic Characteristics		Candida Chromogenic Agar		Total	*p*
		No Growth n (%)	Fungal Growth Present n (%)		
Sex	Male	60 (96.8%)	2 (3.2%)	62 (100.0%)	0.172
	Female	87 (100.0%)	0 (0.0%)	87 (100.0%)	
Total		147 (98.7%)	2 (1.3%)	149 (100.0%)	
Year of	Fourth year	49 (100.0%)	0 (0.0%)	49 (100.0%)	0.126
study	Fifth year	47 (95.9%)	2 (4.1%)	49 (100.0%)	
	Sixth year	51 (100.0%)	0 (0.0%)	51 (100.0%)	
Total		147 (98.7%)	2 (1.3%)	149 (100.0%)	

**Table 4:** Blood agar microbiological isolates in the group of working professionals (dentists).

Demographic Characteristics		Blood Agar			Total n (%)	*p*
		No Growth n (%)	Growth < 10^5^ CoNS n (%)	Growth Eosine-Methylen Blue Agar ≥ 10^5^ CoNS n (%)		
Sex	Male	17 (51.5%)	9 (27.3%)	7 (21.2%)	33 (100.0%)	0.409
	Female	28 (65.1%)	10 (23.2%)	5 (11.6%)	43 (100.0%)	
Total		45 (59.2%)	19 (25.0%)	12(15.8%)	76 (100.0%)	
Year of	Up to 5 years	17 (77.3%)	5(22.7%)	0 (0.0%)	22 (100.0%)	0.139
experience	5–15 years	16 (55.2%)	7 (24.1%)	6 (20.7%)	29 (100.0%)	
	Over 15 years	12 (48.0%)	7 (28.0%)	6 (24.0%)	25 (100.0%)	
Total		45 (59.2%)	19 (25.0%)	12 (15.8%)	76 (100.0%)	

**Table 5:** Microbiological isolates on еosin-methylene in the group of working professionals (dentists).

Demographic Characteristics		Eosin-Methylene Blue Agar			Total n (%)	*p*
		No Growth n (%)	Growth < 10^5^ n (%)	Growth ≥ 10^5^ n (%)		
Sex	Male	26 (78.8%)	6 (18.2%)	1 (3.0%)	33 (100.0%)	0.156
	Female	40 (93.0%)	3 (7.0%)	0 (0.0%)	43 (100.0%)	
Total		66 (86.8%)	9 (11.8%)	1 (1.3%)	76 (100.0%)	
Year of	Up to 5 years	21 (95.5%)	1(4.5%)	0 (0.0%)	22 (100.0%)	0.069
experience	5–15 years	22 (75.9%)	7 (24.1%)	0 (0.0%)	29 (100.0%)	
	Over 15 years	23 (92.0%)	1 (4.0%)	1 (4.0%)	25 (100.0%)	
Total		66 (86.8%)	9 (11.8%)	1 (1.3%)	76 (100.0%)	

**Table 6:** Microbiological isolates on chrom agar in the group of working professionals (dentists).

Demographic Characteristics		Candida Chromogenic Agar			Total n (%)	*p*
		No Growth n (%)	Yeast Growth Present n (%)	Mold Growth Present n (%)		
Sex	Male	29 (87.9%)	2 (6.1%)	2 (6.1%)	33 (100.0%)	0.064
	Female	43(100.0%)	0 (0.0%)	0 (0.0%)	43 (100.0%)	
Total		72 (94.7%)	2 (2.6%)	2 (2.6%)	76 (100.0%)	
Year of	Up to 5 years	19 (86.4%)	2 (9.1%)	1 (4.5%)	22 (100.0%)	0.185
experience	5–15 years	28 (96.6%)	0 (0.0%)	1 (3.4%)	29 (100.0%)	
	Over 15 years	25(100.0%)	0 (0.0%)	0 (0.0%)	25 (100.0%)	
Total		72 (94.7%)	2 (2.6%)	2 (2.6%)	76 (100.0%)	

**Table 7:** Blood agar microbiological isolates in the two groups.

Demographic Characteristics		Blood Agar			Total n (%)	*p*
		No Growth n (%)	Growth < 10^5^ CoNS n (%)	Growth 10^5^ CoNS n (%)		
Sex	Male	59 (62.1%)	27 (28.4%)	9 (9.5%)	95 (100.0%)	0.096
	Female	98 (75.4%)	23 (17.7%)	9 (6.9%)	130 (100.0%)	
Total		157 (69.8%)	50 (22.2%)	18 (8.0%)	225 (100.0%)	
Group	Students	112 (75.2%)	31 (20.8%)	6 (4.0%)	149 (100.0%)	0.004
	Working professionals	45 (59.2%)	19 (25.0%)	12 (15.8%)	76 (100.0%)	
Total		157 (69.8%)	50 (22.2%)	18 (8.0%)	225 (100.0%)	

**Table 8:** Microbiological isolates on eosin-methylene blue in the two groups.

Demographic Characteristics		Eosin-Methylene Blue			Total n (%)	*p*
		No Growth n (%)	Growth < 10^5^ n (%)	Growth > 10^5^ n (%)		
Sex	Male	79 (83.2%)	15 (15.8%)	1 (1.1%)	95 (100.0%)	0.016
	Female	123(94.6%)	7 (5.4%)	0 (0.0%)	130 (100.0%)	
Total		202(89.8%)	22 (9.8%)	1 (0.4%)	225 (100.0%)	
Group	Students	136(91.3%)	13 (8.7%)	0 (0.0%)	149 (100.0%)	0.277
	Working professionals	66 (86.8%)	9 (11.8%)	1 (1.3%)	76 (100.0%)	
Total		202(89.8%)	22 (9.8%)	1 (0.4%)	225 (100.0%)	

**Table 9:** Microbiological isolates on Candida chromogenic agar in the two groups.

Demographic Characteristics		Candida Chromogenic Agar			Total n (%)	*p*
		No Growth n (%)	Yeast Growth Present n (%)	Mold Growth Present n (%)		
Sex	Male	89 (93.7%)	4 (4.2%)	2 (2.1%)	95 (100.0%)	0.015
	Female	130(100.0%)	0 (0.0%)	0 (0.0%)	130 (100.0%)	
Total		219 (97.3%)	4 (1.8%)	2 (0.9%)	225 (100.0%)	
Group	Students	147 (98.7%)	2 (1.3%)	0 (0.0%)	149 (100.0%)	0.107
	Working professionals	72 (94.7%)	2 (2.6%)	2 (2.6%)	76 (100.0%)	
Total		219 (97.3%)	4 (1.8%)	2 (0.9%)	225 (100.0%)	

## Data Availability

All data collected in this study were anonymized prior to analysis to ensure participant confidentiality. Public access to the dataset is restricted for ethical reasons; however, the data are available upon reasonable request from the corresponding author.

## Abbreviations

The following abbreviations are used in this manuscript:

CoNS	Coagulase-negative staphylococci
CFU	Colony-Forming Unit
